# Chronic migraine long-term regular treatment with onabotulinumtoxinA: a retrospective real-life observational study up to 4 years of therapy

**DOI:** 10.1007/s10072-020-04283-y

**Published:** 2020-02-12

**Authors:** Antonio Santoro, Massimiliano Copetti, Anna M. Miscio, Maurizio A. Leone, Andrea Fontana

**Affiliations:** 1 Unit of Neurology, Fondazione IRCCS Casa Sollievo della Sofferenza, San Giovanni Rotondo, FG Italy; 2 Unit of Biostatistics, Fondazione IRCCS Casa Sollievo della Sofferenza, San Giovanni Rotondo, FG Italy

**Keywords:** Chronic migraine, Headache, OnabotulinumtoxinA, Long-term treatment, Real-life, Botox®

## Abstract

**Introduction:**

OnabotulinumtoxinA (BoNT-A) was proved effective and safe in chronic migraine (CM) prevention by the Phase III Research Evaluating Migraine Prophylaxis (PREEMPT) and Phase IV Chronic migraine OnabotulinuMtoxinA Prolonged Efficacy open-Label (COMPEL) trials over 1 and 2 years of treatment, respectively. Real-life studies highlighted BoNT-A sustained benefits up to 3 years of administration. Aim of this retrospective real-life study was observing within a 4-year timeframe the progress of a consecutive series of CM patients treated with BoNT-A and evaluating whether long-term quarterly treatment (up to 16 cycles) confirms the outcomes of previous studies over shorter periods of therapy.

**Methods:**

One hundred nine chronic migraineurs were quarterly treated with BoNT-A according to the PREEMPT paradigm. Headache days and hours, analgesics intake and latency time together with disability were analysed at baseline, thereafter bi-annually up to 48 months. Patient responsiveness (improvement in monthly headache days and hours versus baseline) was computed at each study timepoint.

**Results:**

A significant overall decrease from baseline to the 48-month assessment (*p* < 0.001) was evidenced for the mean number of monthly headache days and hours, analgesics intake and latency time. Severe disability cases significantly decreased at 6 months (*p* < 0.001), and a progressive shift towards lower degrees of disability was observed at each subsequent timepoint. A gradual percentage increase of responsive cases was observed as treatment was repeated over time. Transitory neck pain was reported in 6 cases.

**Conclusions:**

This study appears to reconfirm the benefits of long-lasting CM prevention with BoNT-A, thus supporting quarterly treatment with BoNT-A over several year.

## Introduction

Chronic migraine (CM) is a complex neurological disorder constituting one of the ten leading causes of disability worldwide [1]. Each year approximately 2.5% of patients with episodic migraine develop new onset CM [2]. Compared with episodic migraineurs, CM patients more likely suffer also from depression, anxiety and other co-morbidities [3], which can act on migraine incrementing disability and an uncontrolled multiple need of analgesics, thus leading to medication overuse headache (MOH) as a complication of CM [4]. Abortive medications are overused in around 50–80% of chronic migraineurs [5] and are associated with a greater risk of adverse events (AE) [6, 7]. Therefore, preventing CM is crucial not only to lessen the frequency and severity of migraine attacks, but also to reduce the need of analgesics.

The efficacy and safety of onabotulinumtoxinA (BoNT-A) for the prevention of CM was established in the PREEMPT trials [8–11] demonstrating that repeated treatment with BoNT-A (≤ 5 injection cycles) could safely produce significant improvements in headache symptoms, analgesics intake and health-related quality of life in chronic migraineurs. Thereafter the phase IV COMPEL study expanded these results in migraineurs who received up to nine BoNT-A administrations over 2 years [12].

Clinical trial findings have been corroborated by several real-world studies. The efficacy of quarterly repeated BoNT-A cycles was shown beyond the first year of treatment in chronic migraineurs with MOH [13]. We proved the efficacy and safety of six quarterly cycles over a period of 18 months in 47 patients with CM, highlighting that efficacy significantly increases upon repeated administration [14]. Other real-life studies confirmed BoNT-A safety and effectiveness for periods of two [15–19] and three [20, 21] years, evidencing the sustained therapeutic benefits associated with long-term treatment. Moreover, a general worsening of patient conditions was reported upon abrupt discontinuation of treatment [13] or prolongation of the inter-injection time interval [22]. And as a result, BoNT-A is considered a long-lasting therapy for CM prevention by a remarkable number of clinicians [23].

With this in mind, we conducted the present retrospective study to analyse in real-life within a 4-year timeframe the progress of a cohort of chronic migraineurs regularly treated with BoNT-A and assess whether long-term term quarterly treatment (up to 16 cycles) reaffirms the sustained benefits and good tolerability reported by previous studies over shorter treatments.

## Methods

### Patients and study design

From March 2013 to April 2019 a total of 399 patients with CM received at least one cycle of BoNT-A at our centre, Fondazione IRCSS Casa Sollievo della Sofferenza, San Giovanni Rotondo, S.C. di Neurologia. Among the whole patient population, this retrospective study includes the consecutive series of patients who received the first treatment by 31 March 2015, thus potentially having a 4-year timeframe for treatment and follow-up when database was locked (31 March 2019). Included patients were adult males and females diagnosed as chronic migraineurs [24] with or without medication overuse, had tried and failed other migraine prophylactics, had received at least one BoNT-A treatment and had at least one usable post-treatment assessment. Exclusion criteria were pregnancy or breastfeeding, symptoms of psychiatric disease and any history of botulinum toxin use for other clinical purposes. To fully reflect a real-life population, patients receiving any preventive or symptomatic therapy were not excluded from this analysis.

The study was carried out in accordance with the Declaration of Helsinki and was approved by the Ethics Committee of IRCCS Istituto Tumori Giovanni Paolo II of Bari at Fondazione IRCCS Casa Sollievo della Sofferenza of San Giovanni Rotondo ICF: VI.0_07 APR 2015. Each patient signed an informed consent for the analysis and publication of data.

All data entered in the database were double-checked vs paper records to ensure all entries were correct and up-to-date. Phone interviews were attempted on patients who had discontinued treatment for unknown reasons.

### Treatment

BoNT-A (BOTOX®; Allergan plc, Ireland) was administered every 3 months (± 10 days) in a day-hospital setting, following the PREEMPT paradigm (155–195 U, in 31–39 sites) [25]. In presence of CM with medication overuse, as defined by ICHD 3rd edition [24], detoxification with intramuscular betamethasone for 6 days was performed alongside the start of BoNT-A prophylactic treatment. After 15 days from detoxification patients were advised to take a maximum of 2 dosage units of NSAIDs (nonsteroidal anti-inflammatory drugs) per week.

### Clinical assessment and outcome measures

The number of monthly headache days, cumulative hours of headache in headache days, abortive medication intakes (total number of dosage units) and related latency time (in hours) were daily recorded by patients on headache diary, then evaluated by the investigator at each quarterly visit. Patient quality of life, expressed as migraine-related disability, was evaluated using the Migraine Disability Assessment (MIDAS) questionnaire [26] that was administered to the patient at baseline and at each quarterly visit to explore disability during the previous 3 months. Baseline values for all parameters were referred to the month preceding the initiation of BoNT-A treatment.

### Statistical analyses

All subjects that received first treatment by 31 March 2015 and have at least one usable post-treatment assessment were included in the analysis. Subjects who discontinued treatment or were lost to follow-up were included up to the point of treatment discontinuation or last known post-treatment assessment.

Outcome measures were analysed bi-annually until the 48-month visit (i.e. after 16 injection cycles), in terms of changes in monthly days and hours of headache, acute medication consumption and latency time, and MIDAS grade distribution with respect to the baseline visit (T0) and to each previous timepoint. At the same timepoints patient responsiveness to BoNT-A treatment, expressed as percentage reduction of the number of headache days and hours with respect to baseline, was also computed to analyse over time patient distribution across four groups of increasing responsiveness, ranging from non-responders (< 30% reduction) to partial responders (≥30 and < 50% reduction), responders (≥ 50% and ≤ 75% reduction) and high responders (> 75% reduction), as previously described [14]. All continuous variables were expressed as mean ± standard deviation (SD), median along with interquartile range (IQR) and range (minimum-maximum), whilst categorical variables were expressed as frequencies and percentages. Changes of outcome measures and patients’ responsiveness over time were assessed by hierarchical generalised linear models (HGLMs) which included the follow-up time as main covariate, assuming Poisson and binomial distribution for continuous and categorical outcomes, respectively. The first-order autoregressive covariance structure was used to account for the correlation between repeated measurements over time. Estimated means (or percentages for categorical variables) were carried out from HGLMs and were reported along with their 95% confidence interval (95% CI), including the follow-up time variable into HGLMs as categorical covariate. The overall difference over time was assessed by looking at the significance of the type III test, whereas pairwise comparisons were assessed as statistical contrasts and *p* values were adjusted following Hochberg’s step-up procedure. The presence of linear trend was assessed by looking at the statistical significance of the regression coefficient estimated for the follow-up time variable when this was included into the HGLMs as continuous covariate. Longitudinal plots of the estimated means over time and histograms of the estimated percentages were further reported, along with error bars which represented 95% CI. Two-sided *p* values < 0.05 were considered for statistical significance. All analyses were performed using SAS Software, Release 9.4 (SAS Institute, Cary, NC, USA), and plots were produced by the computing environment R (R Development Core Team 2008, version 3.6, packages: ggplot2, gridExtra).

## Results

### Demographics, discontinuations and drop-outs

**Fig. 1:**
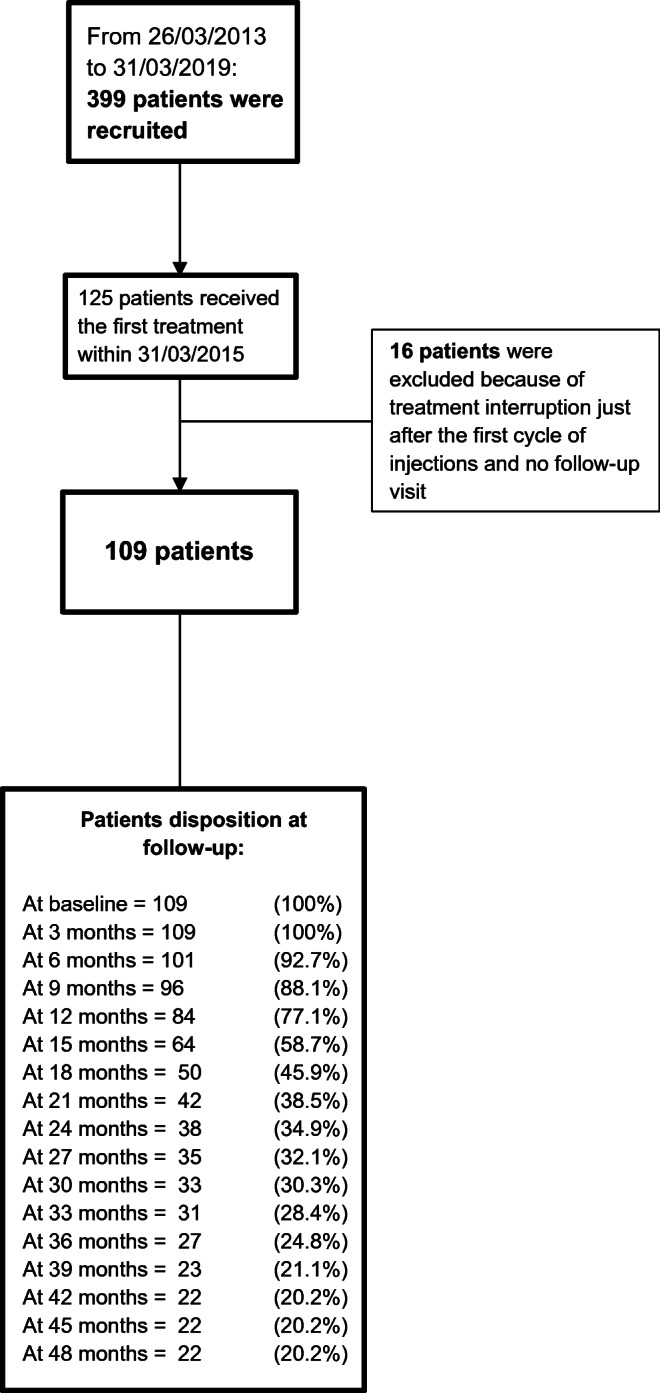
Flow diagram reporting the numbers of individuals at each stage of the study

A consecutive series of 125 subjects started BoNT-A treatment by 31 March 2015. Sixteen patients quitted just after the first treatment. We tracked down twelve of them by phone and found that the reasons for discontinuation were concurrent pathologies preventing patient from coming back to our centre, relocation to another town, discomfort with forehead immobility or number of injections, and reclassification of migraine as secondary. Since no post-treatment assessment was available for these 16 patients, they were excluded for the present analysis as in no way they could contribute to achieving the study objective. Therefore, our study cohort consisted of 109 patients (82 females, 75.2%) with a mean age of 48.1 ± 13.5 years (range 18–76). Symptomatic drug withdrawal cycles were attempted in 20 patients (18.3%) alongside the start of BoNT-A treatment. Patient characteristics at baseline are summarised in Table 1. Within the study cohort BoNT-A treatment was stopped based on clinical judgement in 43 cases who reached the headache frequency threshold we had established for discontinuation. This was ≤ 4 headache days per month for 3 months in the beginning. Thereafter, we decided to set a higher threshold (≤ 4 headache days for 12 months) in order to prevent the risk of relapse after treatment interruption. Worsening was actually reported by 12 patients who came back to our centre after a time period ranging from 5 to 48 months from the interruption. Several discontinuations summarily defined as “lost to follow-up” in our previous study [14] could be better described after the mentioned database audit and phone interviews, which enabled us to identify the reason for discontinuation in a number of cases, as shown in Table 2. Overall, 29 patients were lost to follow-up, resulting in a study drop-out of 29/109 (26.6%). However, twenty-six of them had achieved a reduction of headache days ≥ 50% at the last available follow-up with respect to baseline, whilst 2 (1.8%) were not responding to BoNT-A treatment. Patient disposition through the study is showed in Fig. 1, whilst all treatment discontinuations that occurred before the 16th cycle are outlined in Table 2.

**Table 1:** Demographic details of study cohort at baseline (109 patients)

Age (years)	Mean ± SD Median (IQR) Range	48.1 ± 13.5 48 (39–58) 18–76
Gender (females)	*n* (%)	82 (75.2)
Years of chronic headache	Mean ± SD Median (IQR) Range	11.7 ± 9.8 10 (5–15) 1–60
Patients assuming NSAIDs	*n* (%)	72 (66.1)
Patients assuming triptans	*n* (%)	46 (42.2)
Patients assuming other drugs	*n* (%)	36 (33.0)
Patients assuming other preventive treatments	*n* (%)	47 (43.1)

*SD*, standard deviation; *IQR*, interquartile range (i.e. first-third quartiles); *NSAIDS*, nonsteroidal anti-inflammatory drugs

**Table 2:** Detailed patient disposition within study cohort (109 patients) with respect to treatment discontinuation over time

**Assessment**		**T0**	**T6**	**T12**	**T18**	**T24**	**T30**	**T36**	**T42**	**T48**	
**Ongoing patients** ***n*** **(%)**		109	101 (92.7)	84 (77.1)	50 (45.9)	38 (34.9)	33 (30.3)	27 (24.8)	22 (20.9)	22 (20.2)	
											**Total**
**Discontinuers** ***n*** **(%)**			8 (7.3)	17 (15.6)	34 (31.2)	12 (11.0)	5 (4.6)	6 (5.5)	5 (4.6)		87 (79.8)
**Reasons for discontinuation,** ***n*** **(%)**	Achievement of threshold set by clinician *n* (%)			6 (5.5)	19 (17.4)	9 (8.3)	1 (0.9)	3 (2.8)	5 (4.6)		43 (39.4)
	Pregnancy *n* (%)			1 (0.9)	1 (0.9)			1 (0.9)			3 (2.8)
	Concurrent pathology *n* (%)				1 (0.9)		3 (2.8)				4 (3.7)
	Improvement not in line with patient expectations *n* (%)		1 (0.9)		2 (1.8)	1 (0.9)					4 (3.7)
	Satisfactory status perceived by patient *n* (%)				1 (0.9)						1 (0.9)
	Financial limitations *n* (%)			1 (0.9)							1 (0.9)
	Secondary migraine *n* (%)		1 (0.9)								1 (0.9)
	Lack of efficacy *n* (%)			1 (0.9)							1 (0.9)
	Lost to FU (responders and high responders at the last available FU) *n* (%)		5 (4.6)	7 (6.4)	9 (8.3)	2 (1.8)	1 (0.9)	2 (1.8)			26 (23.9)
	Lost to FU (partial responders at the last available FU) *n* (%)				1 (0.9)						1 (0.9)
	Lost to FU (non-responders at the last available FU) *n* (%)		1 (0.9)	1 (0.9)							2 (1.8)

Percentages were calculated on the total of 109 patients. “High responders” (> 75% reduction), “responders” (≥ 50 and ≤ 75% reduction), “partial responders” (≥ 30 and < 50% reduction) and “non-responders” (< 30% reduction)

### Clinical outcomes

**Fig. 2:**
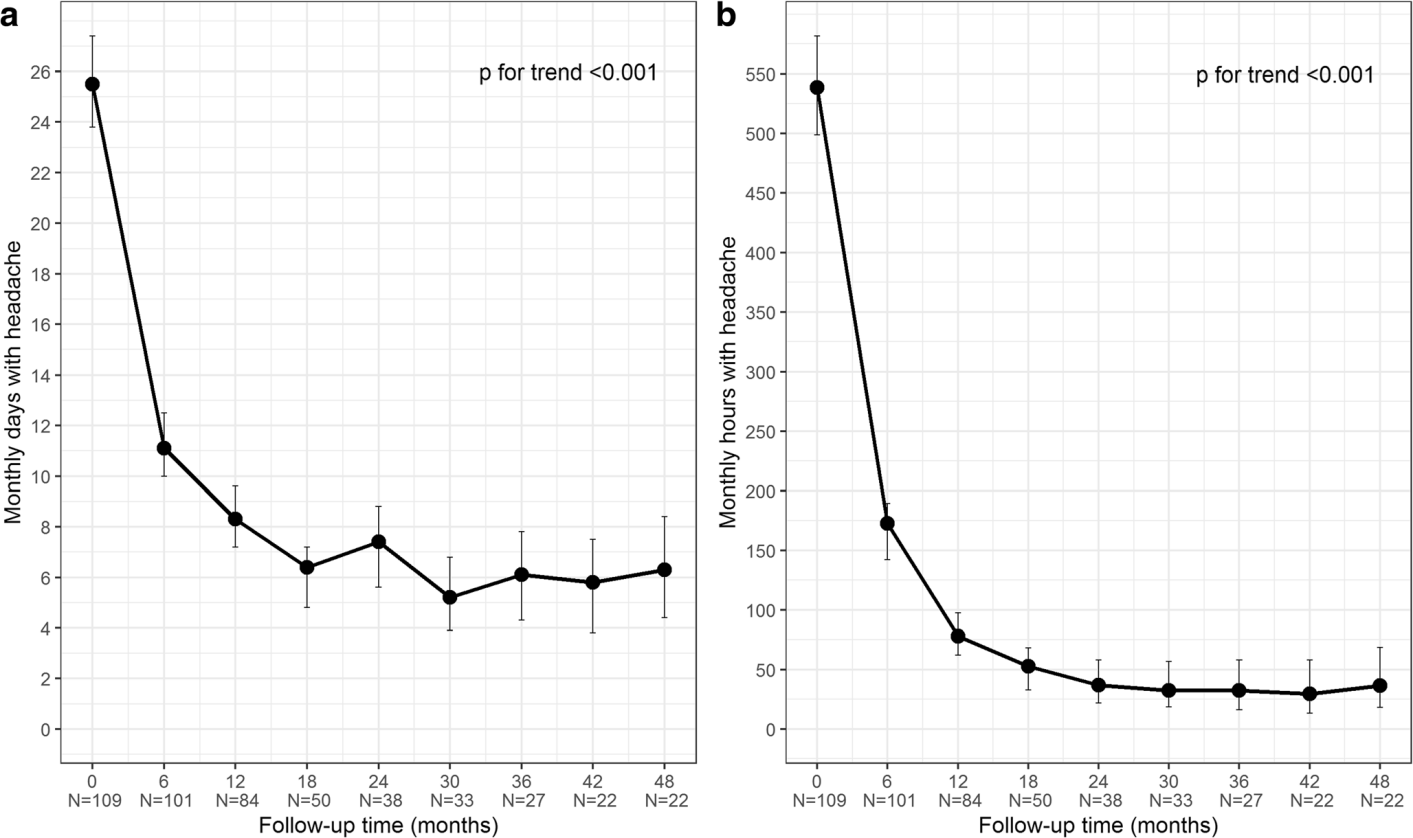
Plots of monthly headache days (**a**) and hours (**b**) means at baseline (T0) up to 48 months (T48), at 6-month time intervals. *Error bars* represent 95% confidence interval around estimated means

Mean numbers of headache days and hours per month at each individual timepoint are reported in Table 3. Mean number of monthly headache days showed a significant overall decrease (*p* for trend < 0.001) from 25.5 ± 5.8 at baseline assessment (T0) to 6.3 ± 3.3 at the 48-month assessment (T48) after the 16th BoNT-A cycle. A statistically significant decrease of monthly headache days was observed at the 6-month assessment (T6) vs T0 (*p* < 0.001), at 12 months (T12) vs T6 (*p* < 0.001) and at 18 months (T18) vs T12 (*p* = 0.004). Thereafter, the mean number of monthly headache days at each individual timepoint was always lower than T0, but not systematically lower than previous timepoint, and pairwise comparisons (each timepoint vs previous one) did not reveal statistically significant differences. Similar changes were observed for the mean number of monthly hours with headache, which significantly decreased over time from 538.6 ± 176.1 at T0 to 36.4 ± 29.0 at T48 (*p* for trend < 0.001) and showed statistically significant pairwise comparisons at the same timepoints, i.e. T6 vs T0 and T12 vs T6 (*p* < 0.001), and T18 vs T12 (*p* = 0.034). At each timepoint, the mean number of monthly hours with headache was always lower than T0 and lower than previous timepoint, with the only exception of T48 vs the 42-month assessment (T42). Plots of the estimated means of monthly headache days and hours over time are shown in Fig. 2a, b, respectively.

**Table 3:** Monthly headache days and hours, from baseline (T0) up to 48 months (T48), at 6-month time intervals

		**T0** (*n* = 109)	**T6** (*n* = 101)	**T12** (*n* = 84)	**T18** (*n* = 50)	**T24** (*n* = 38)	**T30** (*n* = 33)	**T36** (*n* = 27)	**T42** (*n* = 22)	**T48** (*n* = 22)
**Monthly days with headache**	*n*	109	101	84	50	38	33	27	22	22
	Mean ± SD	25.5 ± 5.8	11.1 ± 8.6	8.3 ± 6.5	6.4 ± 5.6	7.4 ± 5.2	5.2 ± 4.0	6.1 ± 3.0	5.8 ± 2.5	6.3 ± 3.3
	95% CI	23.6–27.4	10.0–12.5	7.2–9.6	4.8–7.2	5.6–8.8	3.9–6.8	4.3–7.8	3.8–7.5	4.4–8.4
	Median (IQR)	30 (20–30)	9 (4–16)	6.5 (3–12)	5 (3–8)	6 (4–10)	4 (3–6)	5 (4–8)	5.5 (4–7)	6 (4–10)
	Range	15–30	0–30	0–30	0–30	0–25	0–18	2–12	1–11	0–11
**Monthly hours with headache**	*n*	109	101	84	50	38	33	27	22	22
	Mean ± SD	538.6 ± 176.1	162.8 ± 179.0	78.0 ± 91.3	52.8 ± 77.2	36.8 ± 26.5	32.4 ± 34.0	32.3 ± 23.5	29.7 ± 20.4	36.4 ± 29.0
	95% CI	498.6–581.6	142.0–189.4	62.1–97.6	32.7–68.1	21.8–58.1	18.5–56.7	16.4–58.1	13.3–58.0	18.2–68.8
	Median (IQR)	600 (400–700)	100 (30–200)	42.5 (20–100)	25 (15–50)	32.5 (15–48)	20 (10–48)	25 (12–48)	27.5 (10–45)	34 (8–60)
	Range	112–720	0–700	0–400	0–350	0–100	0–150	0–88	4–77	0–100
***p*** values from HGLM and pairwise comparisons*										
	***p*** **for overall difference**	**T6 vs T0**	**T12 vs T6**	**T18 vs T12**	**T24 vs T18**	**T30 vs T24**	**T36 vs T30**	**T42 vs T36**	**T48 vs T42**	***p*** **for trend**
**Monthly days with headache**	< 0.001	< 0.001	< 0.001	0.004	0.507	0.111	0.641	0.641	0.641	< 0.001
**Monthly hours with headache**	< 0.001	< 0.001	< 0.001	0.034	0.885	0.885	0.885	0.885	0.885	< 0.001

*SD*, standard deviation; *CI*, confidence interval; *IQR*, interquartile range (i.e. first-third quartiles); *p values from hierarchical generalised linear model (HGLM) and adjusted following Hochberg’s step-up procedure

**Fig. 3:**
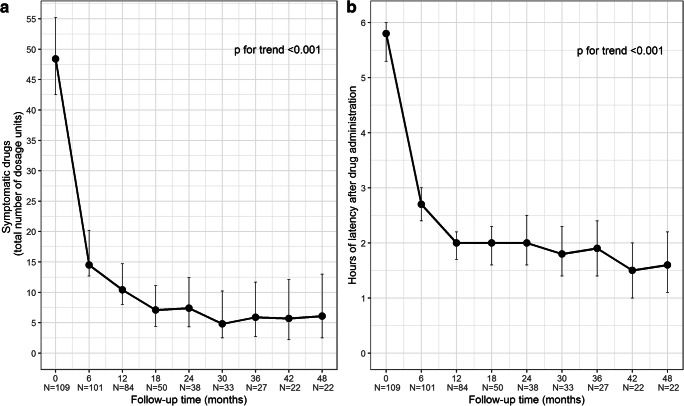
Plots of painkillers used (**a**) and latency time after intake (**b**) means at baseline (T0) up to 48 months (T48), at 6-month time intervals. *Error bars* represent 95% confidence interval around estimated means

In line with the above outcomes, the consumption of painkillers (number of dosage units) significantly dropped over time (*p* for trend < 0.001) from a mean of 48.4 ± 46.4 at T0 to a mean of 6.1 ± 4.1 at T48, showing the sharpest decrease at T6 vs T0 (*p* < 0.001) and another significant decrease at T12 vs T6 (*p* = 0.028). Alongside consumption reduction, analgesics latency time (hours) significantly shrunk from a mean of 5.8 ± 0.9 at T0 to 2.7 ± 1.8 at T6 and 2.0 ± 1.6 at T12 (T6 vs T0 and at T12 vs T6 *p* < 0.001), revealing a significant trend over the entire period under consideration (*p* for trend < 0.001). The changes of analgesics intake and drug response latency time over follow-up are summarised in Table 4 and plotted in Fig. 3a, b, respectively.

**Table 4:** Painkiller consumption (number of dosage units) and latency time after intake (hours), from baseline (T0) up to 48 months (T48), at 6-month time intervals

		**T0** (*n* = 109)	**T6** (*n* = 101)	**T12** (*n* = 84)	**T18** (*n* = 50)	**T24** (*n* = 38)	**T30** (*n* = 33)	**T36** (*n* = 27)	**T42** (*n* = 22)	**T48** (*n* = 22)
**Symptomatic drugs (number of dosage units)**	*n*	109	101	84	50	38	33	27	22	22
	Mean ± SD	48.4 ± 46.4	14.5 ± 22.7	10.4 ± 11.6	7.1 ± 8.0	7.4 ± 6.4	4.8 ± 4.8	5.9 ± 3.3	5.7 ± 3.4	6.1 ± 4.1
	95% CI	42.5–55.2	12.7–20.2	8.0–14.7	4.4–11.1	4.3–12.4	2.5–10.2	2.7–11.7	2.2–12.1	2.5–13.0
	Median (IQR)	34 (20–60)	7 (3–15)	7 (3–12.5)	5 (2–8)	6.5 (4–10)	4 (2–6)	5 (4–8)	5 (4–8)	5 (3–10)
	Range	0–210	0–140	0–70	0–32	0–34	0–24	0–13	0–15	0–13
**Latency time (h)**	*n*	109	101	84	50	38	33	27	22	22
	Mean ± SD	5.8 ± 0.9	2.7 ± 1.8	2.0 ± 1.6	2.0 ± 1.7	2.0 ± 1.6	1.8 ± 1.7	1.9 ± 1.6	1.5 ± 1.0	1.6 ± 1.3
	95% CI	5.3–6.0	2.4–3.0	1.7–2.2	1.6–2.3	1.6–2.5	1.4–2.3	1.4–2.4	1.0–2.0 (1–2)	1.1–2.2
	Median (IQR)	6 (6–6)	2 (1–4)	1 (1–2)	1 (1–2)	2 (1–3)	1 (1–2)	1 (1–2)	1 (1–2)	1 (1–2)
	Range	0–6	0–6	0–6	0–6	0–6	0–6	0–6	0–4	0–5
***p*** values from HGLM and pairwise comparisons*										
	***p*** **for overall difference**	**T6 vs T0**	**T12 vs T6**	**T18 vs T12**	**T24 vs T18**	**T30 vs T24**	**T36 vs T30**	**T42 vs T36**	**T 48 vs T42**	***p*** **for linear trend**
**Symptomatic drugs**	< 0.001	< 0.001	0.028	0.229	0.865	0.865	0.865	0.865	0.865	< 0.001
**Latency time**	< 0.001	< 0.001	< 0.001	0.968	0.968	0.968	0.968	0.968	0.968	< 0.001

*SD*, standard deviation; *IQR*, interquartile range (i.e. first-third quartiles); *p values from hierarchical generalised linear model (HGLM) and adjusted following Hochberg’s step-up procedure

Patient responsiveness to BoNT-A, in terms of improvement in monthly headache days, showed a progressive percentage increase of responsive cases as treatment was repeated over time, with a significant percentage increase from T6 (80.2%) to T12 (92.9%) (T12 vs T6 *p* = 0.029), alongside a mirrored progressive reduction of non-responders, which were totally absent from T30 on. When responsiveness was expressed by reduction of headache cumulative hours, total responders accounted for 88.1% of patients at T6, i.e. after two injection cycles, then significantly increased to represent the whole population under treatment at T12, after the 4th cycle (*p* = 0.002). Distribution of patients into the four responsiveness classes over time is reported in Table 5.

**Table 5:** Distribution of patient response to treatment in terms of reduction of number of days and hours with headache at each study timepoint up to 48 months (T48) with respect to baseline (T0)

		Follow-up (months)								*p* values from HGLM (pairwise comparisons)^#^						
Outcome	Responder groups (% reduction vs baseline^*^)	T6 (*n* = 101)	T12 (*n* = 84)	T18 (*n* = 50)	T24 (*n* = 38)	T30 (*n* = 33)	T36 (*n* = 27)	T42 (*n* = 22)	T48 (*n* = 22)	T12 vs T6	T18 vs T12	T24 vs T18	T30 vs T24	T36 vs T30	T42 vs T36	T48 vs T42
Days with headache	< 30% (non-responders)	20 (19.8)	6 (7.1)	2 (4.0)	2 (5.3)	0 (0.0)	0 (0.0)	0 (0.0)	0 (0.0)	0.029	0.656	0.805	1.000	1.000	1.000	1.000
	≥ 30–< 50%	12 (11.9)	8 (9.5)	4 (8.0)	2 (5.3)	1 (3.0)	0 (0.0)	0 (0.0)	1 (4.5)	0.749	0.749	0.749	0.749	1.000	1.000	1.000
	≥ 50–≤ 75%	32 (31.7)	33 (39.3)	15 (30.0)	16 (42.1)	10 (30.3)	13 (48.1)	10 (45.5)	10 (45.5)	0.670	0.670	0.670	0.670	0.670	1.000	1.000
	> 75%	37 (36.6)	37 (44.0)	29 (58.0)	18 (47.4)	22 (66.7)	14 (51.9)	12 (54.5)	11 (50.0)	0.643	0.075	0.643	0.336	0.643	0.643	0.687
	≥ 30% (all responders)	81 (80.2)	78 (92.9)	48 (96.0)	36 (94.7)	33 (100.0)	27 (100.0)	22 (100.0)	22 (100.0)	0.029	0.656	0.805	1.000	1.000	1.000	1.000
Hours with headache	< 30% (non-responders)	12 (11.9)	0 (0.0)	0 (0.0)	0 (0.0)	0 (0.0)	0 (0.0)	0 (0.0)	0 (0.0)	0.002^§^	–	–	–	–	–	–
	≥ 30–< 50%	9 (8.9)	3 (3.6)	0 (0.0)	1 (2.6)	0 (0.0)	0 (0.0)	0 (0.0)	0 (0.0)	0.279	1.000	1.000	1.000	1.000	1.000	1.000
	≥ 50–≤ 75%	24 (23.8)	14 (16.7)	5 (10.0)	0 (0.0)	2 (6.1)	0 (0.0)	0 (0.0)	0 (0.0)	0.467	0.467	1.000	0.591	1.000	1.000	1.000
	> 75%	56 (55.4)	67 (79.8)	45 (90.0)	37 (97.4)	31 (93.9)	27 (100.0)	22 (100.0)	22 (100.0)	0.001	0.206	0.533	0.533	0.533	1.000	1.000
	≥ 30% (all responders)	89 (88.1)	84 (100.0)	50 (100.0)	38 (100.0)	33 (100.0)	27 (100.0)	22 (100.0)	22 (100.0)	0.002^§^	–	–	–	–	–	–

Number of patients along with column percentages

*This percentage represents the amount of relative responsiveness during the follow-up and was calculated as follows:

[(outcome at baseline − outcome at follow-up)/outcome at baseline] × 100. Based on this percentage, patients were classified as follows:

“Non-responders” (< 30% reduction), “partial responders” (≥ 30 and < 50% reduction), “responders” (≥ 50 and ≤ 75% reduction) and “high responders” (> 75% reduction)

^#^*p* values from hierarchical generalised linear models (HGLM) and adjusted following Hochberg’s step-up procedure; ^§^*p* values from exact McNemar test (HGLMs were not estimable because of degenerate distribution of responders/non-responders over time, i.e. they were all 0% or 100% after the sixth month)

**Fig. 4:**
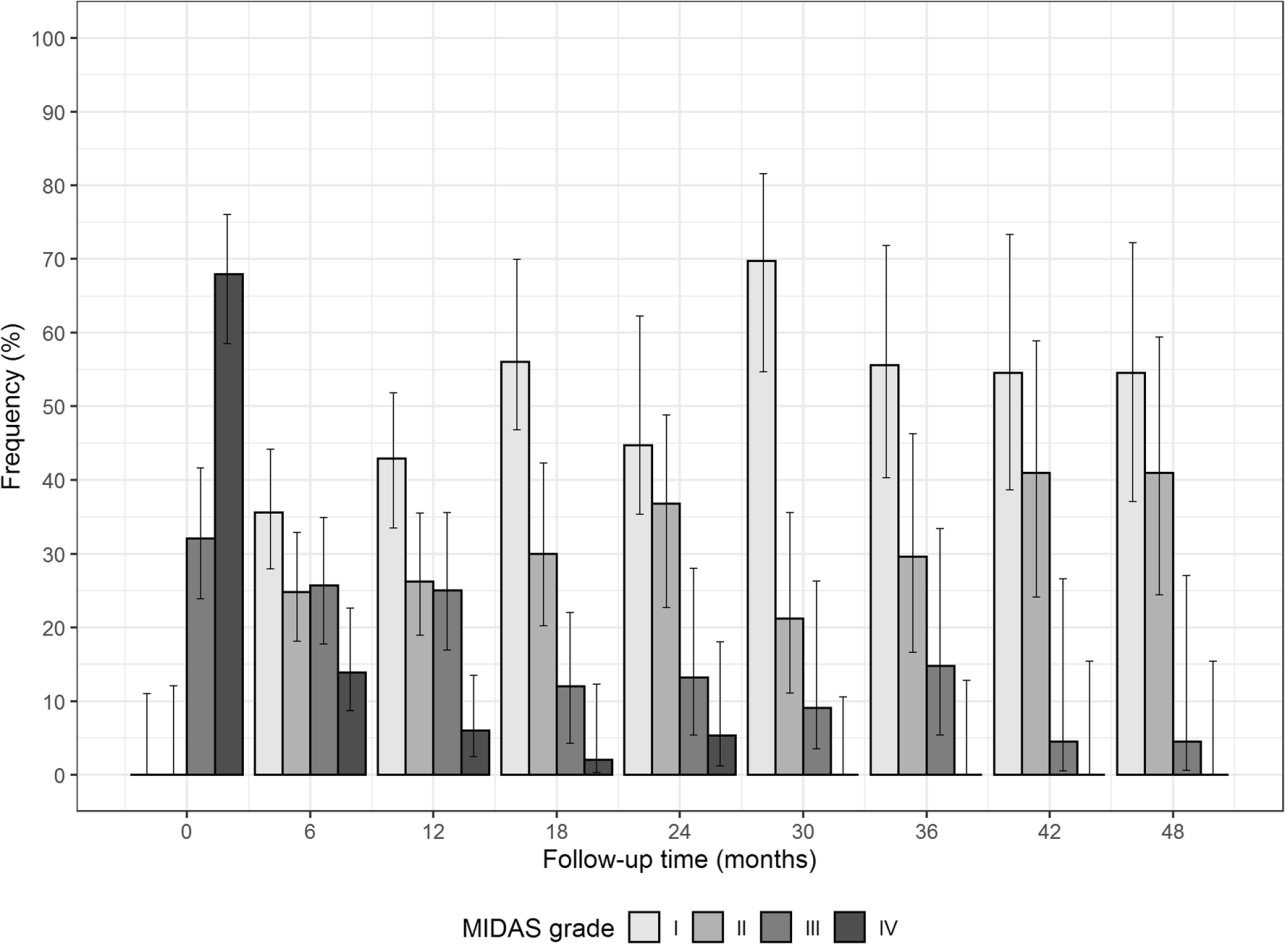
Frequency distribution for MIDAS grade at baseline (T0) and after 48 months (T48), at 6-month time intervals. *Error bars* represent 95% confidence interval around estimated percentages

Patient headache-related disability showed a progressive improvement at each timepoint and over the entire 48-month period, as revealed by MIDAS grade distribution over time (Fig. 4). At T0 patients reported a high degree of disability ranging from Grade III, i.e. moderate disability (32.1%), to Grade IV, i.e. severe disability (67.9%), as shown in Table 6. At T6 severe cases significantly dropped to 13.9% (*p* < 0.001), mild to moderate cases accounted for about half of treated patients, and more than one-third exhibited little or no disability (Grade I, 35.6%). In general, as BoNT-A treatment was repeated over time, a progressive shift towards lower degrees of disability was observed at each subsequent timepoint (*p* < 0.001 for trend).

**Table 6:** Patient distribution as per MIDAS grades, from baseline (T0) up to 48 months (T48) at 6-month time intervals

		**T0** (*n* = 109)	**T6** (*n* = 101)	**T12** (*n* = 84)	**T18** (*n* = 50)	**T24** (*n* = 38)	**T30** (*n* = 33)	**T36** (*n* = 27)	**T42** (*n* = 22)	**T48** (*n* = 22)
**MIDAS–*n*** **(%)**	*n*	109	101	84	50	38	33	27	22	22
	Grade I	0 (0.0)	36 (35.6)	36 (42.9)	28 (56.0)	17 (44.7)	23 (69.7)	15 (55.6)	12 (54.5)	12 (54.5)
	Grade II	0 (0.0)	25 (24.8)	22 (26.2)	15 (30.0)	14 (36.8)	7 (21.2)	8 (29.6)	9 (40.9)	9 (40.9)
	Grade III	35 (32.1)	26 (25.7)	21 (25.0)	6 (12.0)	5 (13.2)	3 (9.1)	4 (14.8)	1 (4.5)	1 (4.5)
	Grade IV	74 (67.9)	14 (13.9)	5 (6.0)	1 (2.0)	2 (5.3)	0 (0.0)	0 (0.0)	0 (0.0)	0 (0.0)
***p*** values from HGLM and pairwise comparisons*										
	***p*** **for overall difference**	**T6 vs T0**	**T12 vs T6**	**T18 vs T12**	**T24 vs T18**	**T30 vs T24**	**T36 vs T30**	**T42 vs T36**	**T48 vs T42**	***p*** **for linear trend**
**MIDAS I**	0.002	0.085	0.586	0.085	0.586	0.085	0.586	0.990	0.990	< 0.001
**MIDAS II**	0.182	0.190	0.966	0.966	0.966	0.738	0.966	0.966	0.966	< 0.001
**MIDAS III**	0.035	0.970	0.970	0.181	0.970	0.970	0.970	0.970	0.970	< 0.001
**MIDAS IV**	< 0.001	< 0.001	0.083	0.358	0.358	1.000	1.000	1.000	1.000	< 0.001

*p values from hierarchical generalised linear model (HGLM) and adjusted following Hochberg’s step-up procedure

### Safety

Few patients (6/109, 5.8%) reported neck pain during the first year of therapy with BoNT-A. This AE was transitory and never caused treatment discontinuation.

## Discussion

A successful migraine preventive therapy should reduce the frequency and burden of attacks, thus stopping drug use escalation and improving patient quality of life whilst causing limited side effects. Moreover, improvements should be stable and long-lasting and this benefit should be the most sought-after goal of such therapy. Several studies have been evaluating BoNT-A use in chronic migraineurs since its approval for CM prevention, which was based on PREEMPT results over a 56-week period [11]. PREEMPT findings were then extended by COMPEL study showing persistent clinical benefits during 2 years of therapy [12]. CM prevention with BoNT-A has been further validated by real-world studies reporting a consistently positive trend of improvement beyond the first year of treatment [13], over an 18-month period [14], and over 2 years [15–19]. Two real-life studies on a 3-year treatment [20, 21] reported, on top of good tolerability, continuous improvements of headache frequency [20, 21], migraine severity, headache days with acute medication use [20], pain and analgesics consumption [21], thus supporting the strategy of consistent BoNT-A administration over long time.

A survey revealed that almost 60% of the interviewed clinicians looked at BoNT-A as a long-lasting therapy [23]. This was deemed to reflect practical experience in observing increased benefits over time and rebound worsening upon interruption [27]. A general worsening of patient conditions was indeed reported after therapy discontinuation or prolonged inter-injection time interval even when patient is a stable responder [13, 22]. Nevertheless, the European Headache Federation recommended to stop BoNT-A if patient has less than 10 headache days per month for 3 months [28] and a group of experts suggested to prolong the inter-injection interval based on patient responsiveness [27].

Our study allowed to further explore this highly debated topic. In fact, by observing the progress of a consecutive series of CM patients quarterly treated with BoNT-A within a 4-year timeframe, the study enabled us to evaluate whether the long-term regular treatment (up to 16 injection cycles) confirms the findings of previous studies over shorter periods of therapy.

We assessed BoNT-A efficacy bi-annually over the course of therapy, comparing each timepoint with T0 and previous timepoint. Results confirmed the progressive improvements at each study timepoint compared with T0 for monthly headache days and hours, with an overall significant difference at T48, after the 16th injection session, compared with T0 (*p* < 0.001). The most striking improvements occurred in the first year, as shown in Fig. 2a and b. It is worth to highlight that the significant drop of monthly headache days and hours observed also at T18, which was consistent with our previous study [14], was followed by a more gradual decline and, eventually, by a condition which appears nearly stable over time. This seems to indicate durable efficacy on BoNT-A even after many repeated administrations, as outlined by other authors after 2 [12, 15–19] or 3 [20, 21] years of therapy.

The overall significant reduction of painkillers consumption and latency time (*p* < 0.001) seems to confirm that BoNT-A long-term treatment can also successfully address medication overuse, as suggested by our previous study [14]. In fact, at T48 after 16 treatments analgesics intake and latency time are around one-tenth and one-third of those reported at baseline, respectively. Moreover, the remarkable decrease observed in the first 6 to 12 months was followed by gradual reductions with few fluctuations in the subsequent years. In line with the findings of Guerzoni et al. [21] on BoNT-A use in CM with MOH, these results seem to confirm the stability of BoNT-A effect long after the first year of therapy.

Analysis of responsiveness to BoNT-A confirms that chronic migraineurs who do not have the desired response after the first injection cycle may indeed experience clinical improvement after repeated treatments, as originally highlighted by Silberstein et al. [29]. We observed this “first time response” trend in our previous study focused on a subset of patients that received 6 injection cycles over 18 months [14]. The present study investigated this phenomenon beyond that timepoint in a larger consecutive series of patients over a 4-year period. It should be noted that BoNT-A treatment was consistently administered for several cycles (≥ 4) in patients not responding to treatment but willing to continue it and response started to be observed during the second or the third year of treatment. These cases would have been classified as “lack of response” if we had interrupted treatment after the 3rd cycle or earlier. When responsiveness was expressed as decrease in monthly headache days, our study showed a progressive reduction of non-responders, which disappeared after ten injection cycles, alongside a progressive increase of total responders upon regularly repeated treatment over time.

Efficacy results are fully reflected by the quality of life improvement. Patient distribution through MIDAS Grade [26] revealed a striking improvement at T6, after two cycles, with a significant decrease of severe cases (Grade IV), which thereafter continued to decrease and totally disappeared at T30, after ten treatments. On the other hand, cases with little to no disability (Grade I) and mild disability (Grade II), which were absent at baseline, altogether became more than half of the patients at T6, then progressively increased over time, up to representing 95% of the patients at T48. All in all, the consistent trend of improvement of MIDAS distribution observed in our previous study [14] appears to be extended over 4 years of treatment.

Some limitations of our study are intrinsic to its real-life nature, i.e. our course of action was driven by day-to-day experience and not by an a priori designed study protocol. The decision to stop treatment was indeed not always consistent throughout the study, since our requirements for treatment discontinuation became more stringent over time. Furthermore, at the beginning of our experience, we did not dedicate enough time to talk to our patients as we currently do and, as a result, several patients discontinued treatment, sometimes because disappointed with the outcome. Over the years, by improving our organisation, we have been able to more extensively communicate with our patients, in order to set realistic expectations and explain that CM requires sustained and possibly continuous treatment.

## Conclusions

Our study confirmed that BoNT-A quarterly administration up to 4 years is a viable therapeutic approach in the prevention of CM. On top of eliciting significant overall improvements of chronic migraineurs conditions, BoNT-A looks persistently effective and well tolerated even upon many repeated administrations, appearing thus able to “stabilise” patient in a comfortable status which is maintained over time. Our study also confirmed the risk of relapse upon treatment discontinuation. These findings altogether seem to corroborate the strategy of CM long-lasting prevention through quarterly treatment with BoNT-A over several years.

## Abbreviations

AE: Adverse event
BoNT-A: OnabotulinumtoxinA
CM: Chronic migraine
COMPEL: Chronic migraine OnabotulinuMtoxinA Prolonged Efficacy open-Label
MIDAS: Migraine Disability Assessment
MOH: Medication overuse headache
PREEMPT: Phase III Research Evaluating Migraine Prophylaxis Therapy
